# Becoming women: period. Perceptions of barriers and facilitators to menstrual hygiene management programs for Pakistani girls

**DOI:** 10.3389/fpubh.2023.1083688

**Published:** 2023-08-11

**Authors:** Alexandria Alisa Proff, Surhan Fatima, Mónica Lorena Sánchez Limón

**Affiliations:** ^1^Department of Humanities and Social Sciences, American University of Ras Al Khaimah, Ras Al-Khaimah, United Arab Emirates; ^2^Department of Mathematics and Social Sciences, Sukkur Institute of Business Administration (IBA) University, Sukkur, Pakistan; ^3^Faculty of Commerce and Administration, Autonomous University of Tamaulipas-Mexico, Tamaulipas, Mexico

**Keywords:** Pakistani girls, menstruation, menstrual hygiene management, WASH in Schools, Pakistan

## Abstract

Discussions on and around menstruation are often considered a cultural taboo in many parts of Pakistan. Mostly, individuals avoid discussing menstruation and lack awareness about its related health and hygiene issues. Sexual health education is entangled in a complex myriad of social and cultural stigmas. Limited knowledge and lack of access to menstrual health management (MHM) resources decrease the self-confidence of girl students and lead to reluctance to seek help or advice. This quantitative study aimed to gain a deeper understanding of the intersectionality of the influences leading to barriers and facilitators of access to the MHM program in a public school in the Hyderabad district and utilize this information to construct a framework for evaluation of the Water, Sanitation, and Hygiene (WASH) in Schools program. This study sampled all girls' elementary and higher secondary schools in the Hyderabad district. Due to the limitations of time and access during the global COVID-19 pandemic, the researchers collected data from more than 100 school leaders and teachers regarding the MHM facilities in their schools through an online self-administered survey. The data were then analyzed in SPSS for frequencies, mean scores, and standard deviation. The results suggest that school leaders and teachers of the Hyderabad district perceive MHM facilities to be significantly low in terms of both resources and policies. Schools worldwide are introducing life skills, hygiene, good health, and wellness subjects. Pakistan needs to change its educational policy for the welfare of women, who comprise a magnanimous 48.54% of its population. Moreover, with the planning and implementation of programs such as WASH in Schools (WinS), the perceptions of barriers and facilitators to MHM facilities in Pakistan must be studied to fight the taboo and raise awareness about the same.

## 1. Introduction

Talking about menstruation equates to shamelessness in Pakistani society. “In countries like Pakistan, where impositions on any sort of dialogue are strongly influenced by religious and traditional practices, public dialogues focusing on sexuality education are considered extremely taboo and invite great criticism and outrage from the public” (1). Due to this mindset, people avoid talking about menstruation and lack awareness about health and hygiene issues related to it.

In the National Curriculum Framework of Pakistan (2), the mention of sexual health or menstruation health management is non-existent. The only health education mentioned in the National Educational Policy (3) is “Physical Education, Health and Sports in Education.” Girls in Pakistan get to know about menstruation from their mothers or close relatives. A study of understanding puberty and health-related problems among adolescents in Karachi revealed many misconceptions about menstruation such as the “majority (98%) of the participants had received this information from their mothers who had also told them to avoid bathing (70%), praying or preaching (90%), carrying heavy weight (89%), and eating eggs, beef, and fish during menstruation. Remaining participants reported receiving information from elder sister, or a friend” (4).

Talpur and Khowaja (5) also credit a qualitative study on adolescents which reported that “girls perceived menstruation as the ability to give birth; and that having a shower during menstruation periods is harmful for their health”. Such perceptions are due to a lack of formal education and inadequate resources to seek knowledge about it.

Scarce research is available on the topic of sexual health and menstruation hygiene education in Pakistan. But, the available limited research strongly advocates the need to have curriculum-based education in our academic institutions. Research highlights an increased level of dissatisfaction among the young with the available health services in Pakistan (5). Shaikh and Ochani (1) also emphasize that “the impact of having sexuality education cannot be overstated, especially in a country like Pakistan, where it can help tackle multiple problems at once”.

The UN Sustainable Goals 4 and 6 address the importance of clean water, sanitation, and equitable quality education for all. Instead of this, Pakistan is internationally committed to focusing on WASH (Water, Sanitation, and Hygiene) throughout the country. UNICEF, with the support of the Government of Pakistan, is actively working to accelerate progress for children, achieve sustainable development goals, and help children realize their rights (6).

To honor the said commitment, UNICEF in consultation with the Ministry of Federal Education and Professional Training initiated the formulation of the WASH in Schools (WinS) Strategy in 2015. After an initial consultative workshop with all stakeholders in October 2016, WASH in School Strategic Plan (2017–2022) was developed. Although the major outcomes are varied, one such outcome regarded the issue of menstrual hygiene management in schools. But, the same “is not prioritized in teacher or student curriculum, neither is it addressed in the school WASH program” (7). The WinS program for Sindh accompanies certain threats and challenges. One major challenge states that “in majority of girls high schools there is inadequate or no provision of Menstrual Hygiene Management (MHM); … and a number of 4,022 schools will require MHM facility and services for students and teachers, to be completed by June 2010” (7).

Currently, certain non-profit and non-government organizations such as Water Aid Pakistan work with UNICEF, UK Aid, and other international organizations on the issues of MHM. Water Aid in collaboration with UK Aid implemented the project in Nepal and Pakistan from November 2014 to March 2018 titled “Ensuring girls' rights through school-based WASH and improved menstrual hygiene management (MHM).” As a result of this large-scale international investment, 106 schools in Punjab, Pakistan were renovated, and WASH infrastructure was constructed in the same. They also worked on raising awareness about WASH rights and MHM in schools and communities; along with developing sustainable supply chain mechanisms for menstrual hygiene materials.

The project was successful as it mainstreamed MHM in a cost-effective manner and contributed to improving girls' rights, menstrual hygiene management, and raising the level of awareness at the institutional level. In Pakistan, the project effectively reached its target to construct or improve sanitation facilities, including menstrual hygiene and hand washing facilities in 110 schools, 60 in the district of Swat and 50 in the district of Muzaffargarh (8).

However, WASH and MHM facilities in Sindh province have been fairly negligible. Mumtaz et al. (9) conducted a survey on the information needs of adolescent girls regarding MHM in Sindh, identifying “significant information needs, specifically around physiology of puberty and menstruation; recognition and relief of menstrual symptoms; appropriate menstrual hygiene and management practices; and social, physical, religious and dietary restrictions. Moreover, they also concluded that water, sanitation and hygiene facilities in schools are inadequate to meet menstruating girls' needs”. Their study recommends the development of an MHM health education module to be taught as a part of the girls' school curriculum.

Nawaz et al. (10) conducted a cross-sectional study with 314 girls 12–15 years of age from girls' high schools in Hyderabad, Sindh. They assert that 60.2% of girls use cloth during menstruation, and the major reason for absenteeism from school is the fear of stains. They suggest that the ratio of absenteeism can be reduced if proper cleanliness facilities are available for changing absorbents (pad/cloth) in schools. Although the study was conducted with girls currently enrolled in schools, quality menstrual and reproductive education was missing.

The literature suggests the unavailability of MHM facilities in the public schools of Sindh. Although, certain projects have been implemented at small scales in Punjab, KPK, and Balochistan. But, the schools in Sindh province lag far behind. A strategic plan of WASH in Schools (WinS) has been framed by UNICEF, and it plans to cater to the need for MHM programs in schools in Sindh by the end of June 2020.

In light of the above literature, it is evident that a problem exists for young girls in Pakistan, especially in Sindh province. Hence, this study aims to seek answers to the following questions:

1. What are the perceptions of school teachers and leaders about barriers and facilitators to MHM programs in their schools?
2. How does the WASH in Schools (WinS) program facilitate the girl students of grades 6–8 in urban public schools of Sindh, Pakistan?

Health education is a basic right of the young generation. It helps them understand the changes their bodies undergo during puberty. But, sadly, sexual health education is entangled in complex social and cultural stigmas, fears, and misconceptions. Limited knowledge and lack of access to resources decrease their self-confidence, and they become reluctant to seek help or advice (11).

To date, sanitary pads in Pakistan are sold in black plastic bags and not one person says the word “period” out loud. Girls pretend to pray during their “*off* ” days to avoid the shaming eyes of male members of the family. The only information they have about menstruation is either from a mother (who is most likely to be equally misinformed) or a close relative (who they cannot relate to and are not comfortable speaking with). Therefore, it is crucial to have a safe place for girls to learn and discuss a natural process that their bodies go through. They need proper guidance and support during puberty which can be provided by trained teachers/counselors in their schools. The introduction of menstrual hygiene education in Pakistan will need social, cultural, and political reforms at a national level. “Menstrual hygiene management requires solutions grounded in unique local contexts that ensure that girls have comprehensive and accurate information about their bodies, their options, their rights, and are able to make informed decisions about their health” (12).

Schools all around the world are introducing subjects related to life skills, hygiene, good health, and wellness. Personal Universal and Life Skills Education (PULSE) for grades 5–8 in Our Own School, Dubai, is an exclusive example of the same. Pakistan needs to make changes in educational policy and take the first step to play its part in the welfare of women who comprise a magnanimous 48.54% (13) of its population. Moreover, with the planning and implementation of programs such as WASH in Schools (WinS), the perceptions of barriers and facilitators to MHM facilities in Pakistan must be studied to fight the taboo and raise awareness about the same.

## 2. Materials and methods

To find answers to the questions posed in this study, the research is designed with a quantitative approach where survey design is deemed appropriate to study the opinions of people regarding menstrual hygiene management. As defined by Creswell (14) “A survey design provides a quantitative or numeric description of trends, attitudes, or opinions of a population by studying a sample of that population. From sample results, the researcher generalizes or draws inferences to the population” (p. 263).

### 2.1. Population and sample

The study focuses on the MHM facilities provided to girls' students of elementary and higher secondary schools in Sindh. Therefore, all elementary and higher secondary schools in Sindh province of Pakistan, and all girls' students of grades 6–8 are the population of the study. However, due to time and accessibility restrictions, the researchers implemented a multi-cluster sampling approach with convenience sampling of all teachers and school leaders of girls' elementary and higher secondary public schools in the Hyderabad district of Sindh Province in Pakistan. In total, 300 teachers and school leaders were sent the questionnaire, of which 106 responded.

### 2.2. Data collection and instrumentation

Since the study was quantitative in nature, the data were collected using a cross-sectional online self-administered questionnaire. As per the nature and requirement of the study, a quantitative research approach was used to measure the perception of barriers and facilitators to the menstrual hygiene management programs for girls in Sindh Pakistan. Moreover, the survey method was used to collect data using a cross-sectional online self-administered questionnaire. Multiple statistical techniques were used to analyze data, including mean, frequency, and standard deviation. More specifically, this was descriptive, cross-sectional research. The data were collected from nine districts of the Hyderabad Directorate of Education in Sindh province. The sample of the target population was drawn from teachers, staff, and higher management of elementary and higher secondary schools of the Directorate of Hyderabad in Sindh province. A convenience sampling method was used to collect data. The unit of analysis of the study was faculty and management from various departments of the selected schools. The questionnaire was sent to 300 teachers and school leaders via email and their respective official WhatsApp groups, out of which 106 teachers returned the completed survey. The online survey included information notifying participants of the nature of the study and that their participation was entirely voluntary. All participants had the right to withdraw at any time. The study operated under certain delimitations. The study was delimited to all girls' elementary and higher secondary public schools of the Hyderabad Directorate of Education only.

### 2.3. Measures

The research instrument comprised of self-administered questionnaire as the primary source of data collection. A set questionnaire using a 4-point Likert scale was used to measure the responses where 1 indicates strongly agree and 4 indicates strongly disagree with the items. The questionnaire was divided into three sections where the Perception of Students (PS) section contained 13 items, the Perception of School Leaders (PSL) section comprised 11 items, and the Resources, Policies, and Curriculum (RPC) section gathered data measured using 19 items in the questionnaire. The collected data were then divided into seven constructs for comprehensive analysis of the perception of barriers and facilitators of MHM in the sample schools. The constructs of policy were measured with six items, and the construct of access to information was measured with five items. Moreover, the construct of resources was measured with 12 items, curriculum construct was measured with 5, social taboo with 8, improvement with 1, and WASH in Schools with four items. These items were then further contextualized using the results of the one open-ended question from the questionnaire, which asked, “In what ways can Menstrual Hygiene Education by [sic] improved in Schools?” The responses to this question were grouped according to the attributes and then associated with the corresponding constructs. The researchers checked the reliability of the scale of all seven constructs to see the internal consistency of the items measuring the construct and found all the sub-scales reliable.

The Cronbach Alpha values at the pre-test and actual test were above 0.7, making this study reliable. The collected data were analyzed using SPSS software (version 21.0). Then, the normality of data was checked by evaluating the values of skewness and kurtosis of the normality curve, and it was found that all the values were in the acceptable range of skewness and kurtosis which are 2 and 7, respectively. Moreover, the multivariate outliers were checked by using the Mahalanobis distance measuring technique, and the same were removed to clean the data. In addition to that, Confirmatory Factor Analysis (CFA) was performed to check the fitness of data with the model and it was found that all items loaded on their respective factors. The value of Cronbach's alpha was in the range of 0.846, which is above the acceptable level of 0.70 of reliability.

## 3. Results analysis

Primarily, a descriptive analysis using SPSS 21 was conducted to identify the demographics of the participants based on gender, employment status, and work experience; 99% of the respondents were women, and only 1% of the respondents were men. Moreover, 20% of the respondents were school leaders, principals, or vice-principals, whereas, 80% were class teachers, subject teachers, or staff members. It was also observed that 51% of the respondents have been in the workforce for 10 years or less, and the remaining 49% had an extensive experience of more than 10 years in the field of education. The results of these demographics are mentioned in Table 1.

**Table 1:** Demographics.

**Description**	**Frequency**	**Percentage**
**Employment status**		
School leaders/management	21	19.8
Teachers	82	77.4
Missing	3	2.8
Total	106	100
**Experience**		
<10 years	52	49.1
>10 years	50	49.2
Missing	4	3.8
Total	106	100

### 3.1. Menstruation as a social taboo

The initial questions in the survey were posed to understand the respondents' perceptions of menstruation as a social taboo. They were asked questions about how girls' students react to discussions about menstruation in general and in school, in particular. The sample item in this part of the questionnaire was “At school, girls generally feel safe about discussing their menstruation.” The results reveal that menstruation is perceived as a social taboo in our society. Although, more than half of the respondents believed that generally girls feel safe about discussing their menstruation. But, at the same time, they also significantly perceived that girls do not feel comfortable at school during their menstruation days and/or if they suddenly get their period at school. Moreover, they also acknowledged that girls do not feel comfortable discussing menstruation with others and that they mind talking about it in public.

The responses also strengthened the commonly occurring myths about menstruation in our society where girls are asked to take certain actions when they are on their period. In agreement with the item, “There are certain things that girls do when they are on their period. (If yes, please specify in the given space),” the respondents specified in comments that most of the girls are shy, uncomfortable, in pain, fatigued, and restless. The majority of them agreed that girls who are menstruating at school are usually sitting alone uncomfortably, or wearing a long scarf at their waist in case their clothes are stained. They also do not feel comfortable asking for a sanitary napkin or discussing the problem with their teachers. Most of the respondents also confirmed that the students usually skip school on their first day of menstruation or demand to go back home in case they get their period in school. One of the school leaders commented that “*During periods, She* [meaning they] *feel tired all the time, see bloody clumps, discharge, premenstrual syndrome, extreme bloating, heavy periods and painful cramps.”* Another one explained that “*They feel uncomfortable while standing or coming to teacher for asking something.”* A senior teacher, in response to this item, clarified that “*They usually skip classes and complain of lower abdomen pain.”* It was also clarified by a teacher that “*They want to go home. Some girls are absent or on leave due during[sic] their periods.”*

Similarly, significantly agreeing with the item “There are certain things that girls avoid when they are on their period. (If yes, please specify in the given space),” the respondents specified four categories of actions that the girls avoid during menstruation: participation in physical activities, meeting people or social gatherings, class participation and extra-curricular activities, consumption of certain foods, and taking baths.

These responses show the mainly prevalent myths about menstruation that are believed by students and teachers alike. School leaders and teachers claim that “*They avoid physical activities like games, running etc.,”* and “*they seem lazy and avoid to participate [sic] in any activity”. One* school leader claimed that menstruating girls avoid “*Consuming lots of salt leads to water retention, which can result in bloating, sugar, coffee, spicy foods, red meat.”* A class teacher mentioned that they “*Avoid bath in 1st three days & some food that worsen the symptoms of period.”* Another comprehensively stated the list of actions avoided by girls as “*Running, exercise, avoiding sour foods, avoid ladyfingers, avoid taking bath.”* Mentioning their lack of participation in physical activities and class participation, some respondents commented that “*They avoid to play [sic] games and want to sit alone and not actively participate in class activities.”* It was also mentioned that absenteeism becomes common due to menstruation as a respondent explained that “*Yes some of them avoid to come school if they come so they urge teacher to go back home but don't discuss freely [sic].”* Highlighting the common state of mind of menstruating girls, some respondents commented that “*Mostly not taking interest [sic] their studies and remain silent.” “Avoid from [sic] going to public gatherings, avoid discussion on that* [menstruation] *they usually avoid and feel shy.”* These responses strengthen the perception that menstruation is considered a social taboo in our society and that girl students do not feel comfortable during menstruating days in general and at school, in particular. The results of this construct are presented below in Table 2 which shows that the mean score of the Social Taboo construct was 2.7 which, in turn, shows general agreement with the idea that menstruation is a social taboo in Pakistan. The standard deviation of the construct was found to be 0.3.

**Table 2:** Social taboo.

**S. No**.	**Code**	**Item**	**Disagree**	**Agree**
1	PS1N	At school, girls generally feel safe about discussing their menstruation.	40%	60%
2	PS2N	Girls talk to their teachers when they have questions about their bodies.	54%	46%
3	PS3N	There are certain things that girls do when they are on their period (If yes, please specify in the given space).	32%	68%
4	PS4N	There are certain things that girls avoid when they are on their period (If yes, please specify in the given space).	23%	77%
5	PS8N	Girls are comfortable at school during their menstruation.	61.2%	38.8%
6	PS9N	Girls feel worried when they suddenly get their period at school.	8.7%	91.3%
7	PSL2N	Girls do not feel comfortable in talking to others about menstruation.	16.8%	83.2%
8	PSL3N	Nowadays, girls do not mind talking about menstruation openly in public.	31.3%	68.7%

Mean = 2.7; S.D. = 0.30.

### 3.2. Access to information

The next set of questions was focused on measuring the accessibility of information about menstrual hygiene management to students and teachers. The data revealed that there is a general agreement between the respondents that information regarding menstruation hygiene is not provided to students and teachers. The students are neither taught the correct use of sanitary napkins in schools nor introduced to any other significant information about their menstrual health. Moreover, the teachers are also not given any professional development to handle issues related to the menstruation of students. There is no active access to information for students, teachers, staff, or management. The mean score of this construct was found to be 2.4 with a standard deviation of 0.59; this advocates a significant agreement of respondents against this perception. The results are presented below in Table 3.

**Table 3:** Access to information.

**S. No**.	**Code**	**Item**	**Disagree**	**Agree**
1	PS7N	At school, girls are taught about the correct use of sanitary napkins.	53.9%	46.1%
2	PSL4N	School provides access to information about menstruation for girls.	54.2%	46.8%
3	PSL5N	School provides access to information about menstruation for teachers and staff.	60%	40%
4	PSL6N	Professional development of teachers and staff regarding menstruation and its management is ensured at school.	52.7%	47.3%
5	PSL7N	Class and Subject teachers are given special training about menstruation and how to deal with it.	56.8%	43.2%

Mean = 2.4; S.D. = 0.59.

### 3.3. Curriculum

Researchers also aimed to seek answers about the coverage of menstrual hygiene management topics in the school curriculum. The data for this construct was gathered by five items that measured the curriculum coverage of MHM. The data revealed that school leaders and teachers hold a general agreement about the inclusion of MHM in the curriculum, specifically on the topic of hormonal changes in menstruating girls. Whereas, there is a significant perception of respondents that topics such as menstrual hygiene, pain management during menstruation, and psychological changes in menstruating girls are not covered in the school curriculum. The mean score of this construct was found to be 2.6 with a standard deviation of 0.44, which shows an agreement among the respondents over the crucial topic of the inclusion of MHM in the curriculum. The results are presented below in Table 4.

**Table 4:** Curriculum.

**S. No**.	**Code**	**Item**	**Disagree**	**Agree**
1	RPC14N	School curriculum covers the topic of menstruation in detail.	71.3%	28.7%
2	RPC15N	School curriculum covers the topics of menstrual hygiene.	27.9%	72.1%
3	RPC16N	School curriculum covers the topics of pain management during menstruation.	33.7%	66.3%
4	RPC17N	School curriculum covers the topic of psychological changes in menstruating girls.	33.7%	66.3%
5	RPC18N	School curriculum covers the topic of hormonal changes in menstruating girls.	84.5%	15.5%

Mean = 2.6; S.D. = 0.44.

### 3.4. Policies and procedures

It was crucial for this study to understand the current perceptions of school leaders about policies and practices of MHM in the education system, and to inquire about the stakeholders who make these policies, if any. The results show that there is no policy for menstrual hygiene management at all. There is no written policy about a sudden event of menarche in any student, and neither are there any procedures that are followed in case of such events. It was evident from the data that if a student suddenly gets her first period at school, which is mentally and physically discomforting, she is simply sent back to her home. There is no policy or procedure to provide comfort, ease, and support to her from teachers or school leaders. Neither is there any procedure in place that ensures the way a sudden menstruation case may be handled. It is generally perceived that in case of such an emergency, students either have the sanitary materials that they use or are simply sent back home to avoid any inconvenience. It was also revealed from the data that as there are no policies about MHM, and none are made at higher levels, hence, neither school leaders/teachers nor students are a part of policy-making practices. The results highlight the insignificant importance that MHM is perceived to be given to by policy-makers, as it is highly neglected by all. The construct of policy was found to have a mean value of 2.47 and a standard deviation of 0.53. The results are summarized below in Table 5.

**Table 5:** Policy.

**S. No**.	**Code**	**Item**	**Disagree**	**Agree**
1	RPC7N	School has a clear policy about menstruating girls.	62.5%	37.5%
2	RPC8N	School has a clear procedure about first menstruation (menarche) of a student during school hours.	53.5%	46.5%
3	RPC9N	School has a clear procedure about an event of sudden menstruation of a student.	54%	46%
4	RPC10N	Policies are made at higher levels of management (district/city/province).	46.5%	53.5%
5	RPC11N	Principals/Teachers are an integral part of policy making about menstruation.	32.2%	67.8%
6	RPC12N	Students are an integral part of policy making about menstruation.	37.2%	62.8%

Mean = 2.47; S.D. = 0.53.

### 3.5. Resources

One of the major questions that this study posed was about the available facilities and resources for menstrual hygiene management in public schools in Pakistan. The respondents perceived that there is a significant absence of resources for MHM for girl students. On one hand, it was perceived by ~50% of respondents that a clean functional toilet is available in schools, along with the facility of running water, and dustbins to dispose of sanitary materials such as pads at school for menstruating girls. They perceived that sanitary materials such as napkins are easily available to menstruating girls at home; however, the same is not available for them in schools. On the other hand, it was generally agreed upon by the respondents that facilities such as pain medication, comfort rooms, clinics, or any other medical assistance are not available in schools. Approximately 83% of the respondents argued that regular medical checkups are not arranged for menstruating students. Overall, 57% of the respondents believed that schools do not have adequate resources to support girl students during menstruation; 84% of the respondents agreed with the item “School needs more resources to support girls during menstruation (see Table 6). (If yes, please specify in the given space).” The responses to this item have been divided into five categories that are basic facilities, adequate funds, awareness sessions, medication and comfort room, and implementation of the WASH in Schools program.

**Table 6:** Resources.

**S. No**.	**Code**	**Item**	**Disagree**	**Agree**
1	PS5N	Sanitary materials (like sanitary napkins) are easily available to girls at home.	16.7%	83.3%
2	PS6N	Sanitary materials (like sanitary napkins) are easily available to girls at school.	63.7%	36.3%
3	PS11N	Pain medication is available at school for girls during menstruation.	72.1%	27.9%
4	PS12N	A comfort room is available at school for girls during menstruation.	81.6%	18.4%
5	PS13N	A clinic is available at school for girls during menstruation.	92.2%	7.8%
6	RPC1N	School has adequate resources to support girls during menstruation.	56.8%	43.2%
7	RPC2N	A clean and functional toilet is available at school for menstruating girls.	47.7%	52.3%
8	RPC3N	Running water is available at school for menstruating girls.	31.8%	68.2%
9	RPC4N	Dustbins are available to dispose sanitary materials such as pads at school for menstruating girls.	39.8%	60.2%
10	RPC5N	Medical Assistance is available at school for menstruating girls.	79.5%	20.5%
11	RPC6N	School needs more resources to support girls during menstruation (If yes, please specify in the given space).	15.9%	84.1%
12	RPC13N	School has regular medical check-ups for menstruating girls.	82.8%	17.2%

Mean = 2.3; S.D. = 0.45.

It is evident from the comments that the respondents have written in response to the abovementioned item that there is a serious lack of facilities to implement an MHM program. To bring the issue of lack of basic facilities to the researcher's note, a senior principal claimed that “schools needs [sic] each and every thing regarding this (MHM) issue. They don't have single [sic] thing, and they are not able to provide anything to their students”, which again emphasizes the gravity of the concern. Another teacher added that “We are in need of education and awareness about menstruation at schools; sanitary napkins, pain medications, and other resources are deficient as well. WASH guidelines are not implemented.” The comment clarifies that the school teachers know about the WASH in Schools program and their guidelines but the same are not being implemented due to lack of resources. “School should provide menstrual hygiene education to their students, accessible sanitary products, pain relief medicines and adequate sanitary facilities,” asserted another participant. Adding to the same, a respondent from a rural area highlighted the condition of her school by exclaiming “In rural areas, schools are suffer [sic] from basic facilities so a lot of resources needed especially for girls' school.” Some respondents also realized the importance of awareness sessions by stressing that they “should have personal hygiene sessions at school time by time”. The comments also accentuated the lack of funds by claiming that “As we all know the provided resources for this purpose are managed by teachers mostly. It should be provided by govt. in annual fund with transparency.[sic]”

Medication and access to a comfort/common room for the menstruating students were also recorded with comments like “Girls common room with attach toilet should be made compulasry [compulsory] in Middle n high schools,” “Medication and privacy,” and “Seperate [separate] room for rest, material and medicines.” Another respondent shared the condition of her school by mentioning that “My school have not any resources materials and facilities for the menstrual period (MHM).[sic]” Many respondents mentioned the need for a separate toilet with complete accessories such as “covered dustbin, some basic medicines,” “pads, panty in emergency for girl students as students in government schools are poor they can't afford pads expenses,” and “dustbin, clean washroom, sanitary materials, phenyl, surf, soap, panties for changing in case of emergency.” While others mentioned “medical assistance, training and personal hygiene sessions at school” as necessary. These detailed responses to a particular item in the resources construct highlight the poor condition of MHM facilities in public schools of Hyderabad. The following table of resources construct could also be observed to represent the same. The mean for the resources construct was 2.3, and the standard deviation was 0.45.

### 3.6. WASH in schools program

An important question that this research study sought to answer was the involvement of WASH in Schools programs in providing facilities for menstrual hygiene management to public schools in Sindh. This set of questions in the construct of WASH in Schools comprised four items including “I am aware of the WASH in Schools program.” The findings show that 63.2% of respondents knew about the WinS program but only 32% believed that the program has provided any resources to their school (see Table 7). Moreover, ~68 and 54% of the respondents confirmed that the WinS program keeps a check and balance of the provided resources and that it is an essential part of the MHM in their schools, respectively. The mean for this construct was 2.44 with a standard deviation of 0.57. The overall findings of this construct highlight that WASH in Schools program is an essential part of the MHM facilities in schools but the provided facilities by them are extremely inadequate. The facilities can be made extensive to ensure better MHM facilities for menstruating students.

**Table 7:** WASH in schools.

**S. No**.	**Code**	**Item**	**Disagree**	**Agree**
1	PSL8N	I am aware of the WASH in Schools Program.	36.8%	63.2%
2	PSL9N	WASH in Schools Program has provided resources to my school.	67.7%	32.3%
3	PSL10N	WASH in Schools Program keeps a check and balance of the provided resources.	63.2%	36.8%
4	PSL11N	WASH in Schools Program is an essential part of Menstrual Hygiene Management in my school.	54.2%	45.8%

Mean = 2.44; S.D. = 0.57.

### 3.7. Improvement

It is evident from the data that 87.2% of respondents confirmed that menstrual hygiene education can be improved at schools (see Table 8). This is a significant percentage of school leaders and teachers that consider MHM an essential need for menstruating students in their schools. We recorded 58 critical comments as a response to the last item on the questionnaire “In what ways can Menstrual Hygiene Education be improved in schools?” These 58 respondents mentioned various ways in which MHM can be improved in public schools in Hyderabad.

**Table 8:** Improvement.

**S. No**.	**Code**	**Item**	**Disagree**	**Agree**
1	RPC19N	Menstrual Hygiene Education can be improved at school.	12.8%	87.2%

Mean = 3.0; S.D. = 0.63.

Most of the responses mentioned a need for “access to information” and “training for students, teachers, and parents”. A respondent commented that “*Teachers training, awareness program for students, proper guidance, specific reading material for girls,”* and “*Proper awareness programs must be done in order to improve the overall menstrual hygiene.”* As one school leader commented that “*Through awareness, training and specially improve washroom system in govt. schools in priority basis, funds properly used in every area of school then the environment can be changed.[sic]”* Another respondent suggested using performing arts to raise awareness about menstruation for students and parents alike; “*By short videos, by little skits performed by the students, head, and teachers cooperation and interact with patents* [sic]*.”*

Another important area of focus was “curriculum” where they suggested including the topic of menstrual hygiene in science and other subjects. Comments like “*Through knowledge by books, lectures, and technical experts,” “Through Teachers training programs & curriculum should be cover the topics of menstrual hygiene,” “Monthly a class should be held about this,”* and “*By organizing different programs of awareness,”* represent the need to include MHM in the curriculum of public schools to improve the overall situation.

Moreover, “policy-making and its implementation” was suggested to improve the overall condition of MHM. Suggestions about policy-making and its implementation are evident by comments such as “*Making it part of course & integrated policies & procedure in place,” “By Adding topics in curriculum and making policy about this.”*

Some respondents also believed in comprehensive reforms where the issue is improved in all aspects such as the provision of basic facilities, training, curriculum, policy, appropriate use of funds, and awareness among students and teachers. One instance is as follows:

“*There are various areas in which menstrual hygiene education can be improved at schools. A thorough planned scheme needs to be enacted by higher authorities and its proper monitoring and transparency needs to be ensured. There is lack of resources that can be utilized for the safety and comfort of menstruating girls. Despite various govt. initiatives there is lack of sanitary napkins, medications and its safe disposal at schools. There should be proper tuition regarding menstrual hygiene along with necessary providence* [provision] *of menstrual safety kits*.[sic]”

Other respondents wished to break the barriers of social taboos and discuss menstruation openly and freely so that good hygiene may be maintained. One of them commented:

“*Being a woman, mother and being a teacher our first priority is to take interest in this matter, and teach them (girls* 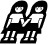
*) about Menstrual periods without any hesitation and freely talk about how to face and how to clean yourself, how to wear proper clothes during the periods. And teach girls about the benefits of periods, Changes of harmones* [hormones], *take proper medicines if girls feel more pain during menstral* [menstrual] *period and take bath with lukewater* [lukewarm water]*, take proper foods and rest. Thanks”*

The following table of improvement construct presents a mean value of 3.0 and a standard deviation of 0.63.

## 4. Discussion on findings

The findings suggest that school leaders and teachers perceive menstruation as a social taboo in Pakistan. Similar findings have been reported in various studies conducted in Pakistan and around the world (15–17). Moreover, it is evident from the findings that menstrual hygiene management is not considered significant in the education system of Pakistan as there is a lack of access to information and training material for students and teachers alike. Similar incidents have been reported in detail in the study by Sommer et al. (18), where the impact of MHM seeps into the opportunities and access to education for menstruating girls around the world. The study also concludes that menstrual hygiene management is neither included in the curriculum nor are there any policies and procedures regarding the same. Mumtaz et al. (17) also advocate similar results through their study, and present that due to the taboo nature of the topic, the same is not included in the curriculum. The menstruating girls are usually unaware of this natural process until menarche, as neither school nor their mothers talk to them about it to seemingly protect the “innocence” of the girls by staying unaware about such things.

A significant perception mentions that there is a dearth of basic resources such as toilets, running water, sanitary materials, pain medication, and medical assistance for menstruating girls in Sindh province. Similar reports are present in the literature where many studies suggest a lack of adequate sanitary material, water supply, disposal mechanism, and a sense of safety and security for menstruating girls around the world including Ghana (19), Kenya (20, 21), Uganda (22), and India and Pakistan (23, 24). In response to the second question, the data confirm that WASH in Schools program is well-known to the schools and is also an essential part of MHM facilities, but the provided resources are significantly low. The literature suggests that various projects have been introduced around the world to facilitate girls' education with special attention to their menstrual hygiene management needs. Some projects aim to reinstall and/or construct WASH infrastructure in schools (8, 25). Much of WASH infrastructure, however, despite a large financial investment, remains non-functional or inadequate (26–28). Finally, this study concludes with the significant agreement of all respondents regarding the need for improvement of MHM facilities in public schools in Sindh. Mumtaz et al. (17) adequately complement this point through their study and affirm that “to be truly effective and to make school environments more enabling and improve girls' engagement during menstruation, current menstrual hygiene management strategies need to address the root causes of poor WASH infrastructure, the issues of governance and corrupt practices. The strategies also need to ensure facility design is sensitive to the gendered and deeply embedded local socio-cultural values and beliefs around menstrual hygiene management” (p. 160).

## 5. Limitations

Given the quantitative nature of this study, there are certain limitations to consider. First, this study did not explore, in-depth, the underlying factors informing the identified barriers and facilitators. Second, this study extended only to Sindh province and did not take into account a sample size generalizable to the entire country. Furthermore, the nature of self-reported data requires a certain degree of skepticism when interpreting the results. Finally, while the results of this study warrant a worthy inclusion into the knowledge of these critical issues, these results do indicate the need for further research and analysis into the underlying influences that inform both barriers and facilitators to menstrual health education in Pakistan.

## 6. Conclusion

This study aimed to study the facilitators and barriers to menstrual hygiene management in public schools in Sindh province. With quantitative research using a cross-sectional online survey, the data were collected from the school leaders and teachers of all elementary and higher secondary girls' public schools of the Directorate of Hyderabad, Sindh. The findings of the study add up to the existing body of literature on menstrual hygiene management in Pakistan and confirm the results of previous studies that the topic of menstrual hygiene management is a social taboo in Pakistan, that the students' and teachers' access to information about the same is limited, the curriculum does not include topics of MHM, that the policy guidelines for MHM practices are not valued/present in the educational system of Sindh, that the WASH infrastructure and resources are very limited and insufficient, and that there is a dire need of improvement in the overall MHM practices in the public schools of Sindh province.

## 7. Recommendations and future research

The following recommendations are made based on the findings of the study:

1. Awareness sessions that discuss menstruation as a natural process should be held by schools for students and parents to break the taboo associated with it.
2. Students' and teachers' access to information regarding menstrual hygiene should be made possible with frequent training, seminars, and workshops.
3. Menstrual hygiene management should be included in the curriculum, and topics such as psychological and hormonal changes in bodies as a result of menstruation shall be discussed freely in classrooms.
4. Policies about MHM should be made and implemented in public schools in Pakistan.
5. School leaders should ensure the implementation of policies and procedures regarding menstruation and menarche to make it less daunting for students to attend school during menstruating days.
6. Basic resources such as separate functional toilets for girls with relevant sanitary materials such as pads, panties, and covered dustbins should be made available in all public schools.
7. WASH in Schools program should be implemented in rural areas of Sindh province as well.
8. All stakeholders should work together to improve the quality of menstrual hygiene management in the public schools in Sindh.

The findings of the study are a single step toward better menstrual hygiene facilities in the public schools in Sindh. Due to the sudden closure of schools in the country owing to the pandemic, the study was limited to online data collection only from one directorate. Hence, further extensive studies in other directorates, provinces, and national scales are suggested for future research. Future studies may also focus on the perception of students and parents to study the overall scope of the problem. Moreover, qualitative studies may also be conducted to understand the underlying reasons for the poor quality of menstrual hygiene management in public schools.

## Data availability statement

The raw data supporting the conclusions of this article will be made available by the authors, without undue reservation.

## Ethics statement

The studies involving human participants were reviewed and approved by Sukkur IBA. The patients/participants provided their written informed consent to participate in this study.

## Author contributions

All authors listed have made a substantial, direct, and intellectual contribution to the work and approved it for publication.
